# Diagnosis of sustainable collaboration in health promotion – a case study

**DOI:** 10.1186/1471-2458-8-382

**Published:** 2008-11-07

**Authors:** Mariken TW Leurs, Ingrid M Mur-Veeman, Rosalie van der Sar, Herman P Schaalma, Nanne K de Vries

**Affiliations:** 1 Youth Department, Netherlands Organization for Health Research and Development, P.O. Box 93245; 2509 AE The Hague, The Netherlands; 2 Health Policy and Economics Department, University Maastricht, PO Box 616, 6400 MD Maastricht, The Netherlands; 3 Health Promotion Department, Netherlands Organization for Health Research and Development P.O. Box 93245; 2509 AE The Hague, The Netherlands; 4 Psychology Faculty, University Maastricht, PO Box 616, 6400 MD Maastricht, The Netherlands; 5 Health Promotion Department, University Maastricht, PO Box 616, 6400 MD Maastricht, The Netherlands; 6 Regional Public Health Service Maastricht, The Netherlands

## Abstract

**Background:**

Collaborations are important to health promotion in addressing multi-party problems. Interest in collaborative processes in health promotion is rising, but still lacks monitoring instruments. The authors developed the DIagnosis of Sustainable Collaboration (DISC) model to enable comprehensive monitoring of public health collaboratives. The model focuses on opportunities and impediments for collaborative change, based on evidence from interorganizational collaboration, organizational behavior and planned organizational change. To illustrate and assess the DISC-model, the 2003/2004 application of the model to the Dutch whole-school health promotion collaboration is described.

**Methods:**

The study combined quantitative research, using a cross-sectional survey, with qualitative research using the personal interview methodology and document analysis. A DISC-based survey was sent to 55 stakeholders in whole-school health promotion in one Dutch region. The survey consisted of 22 scales with 3 to 8 items. Only scales with a reliability score of 0.60 were accepted. The analysis provided for comparisons between stakeholders from education, public service and public health.

The survey was followed by approaching 14 stakeholders for a semi-structured DISC-based interview. As the interviews were timed after the survey, the interviews were used to clarify unexpected and unclear outcomes of the survey as well.

Additionally, a DISC-based document analysis was conducted including minutes of meetings, project descriptions and correspondence with schools and municipalities.

**Results:**

Response of the survey was 77% and of the interviews 86%. Significant differences between respondents of different domains were found for the following scales: organizational characteristics scale, the change strategies, network development, project management, willingness to commit and innovative actions and adaptations. The interviews provided a more specific picture of the state of the art of the studied collaboration regarding the DISC-constructs.

**Conclusion:**

The DISC-model is more than just the sum of the different parameters provided in the literature on interorganizational collaboration, organization change, networking and setting-approaches. Monitoring a collaboration based on the DISC-model yields insight into windows of opportunity and current impediments for collaborative change. DISC-based monitoring is a promising strategy enabling project managers and social entrepreneurs to plan change management strategies systematically.

## Background

Collaborations and partnerships are elemental to health promotion in general and of school health promotion in particular, when promoting healthy life-styles using multi-interventino approaches [1-3]. Intersectoral collaboration where people from different domains, cultures and jargon are expected to work together is not without challenges [4-6]. Interest in the process and prerequisites of collaboration in organizing (school) health promotion, is rising [7,8]. Underlying theories and principles of organization change are widely available from other sectors [9-12]. However, the scientific literature does not provide comprehensive monitoring instruments focusing on the specific pitfalls and opportunities in collaborative processes towards (school) health promotion.

In this article we delineate the DIagnoses of Sustainable Collaboration (DISC) model [13]. The DISC-model is meant to describe the state of affairs of a health promotion oriented collaboration at a certain moment in time, aiming to reveal opportunities and impediments for change. A thorough analysis of the current status of the collaboration supports the selection of suitable change strategies to enhance the development of the collaboration[14].

### Schoolbeat

To illustrate and assess the DISC-model, the 2003 application of the model to the Dutch schoolBeat collaboration is described. The schoolBeat-partners aimed to build a coordinated multi-organization strategy supporting tailored whole-school health promotion [15]. The development of schoolBeat commenced in 2001 when five regional health-promoting agencies joined forces in the south of the Netherlands. The five key-players came from the areas of addiction, mental health, public health, youth care and social welfare [16]. With the recruitment of a researcher and project manager, financed by a national four-year grant by the Healthy Living program of the Netherlands Organization for Health Research and Development, the project advanced in Spring 2002 [15]. This schoolBeat-strategy is based on sharing whole-school health promotion advisory tasks between organizations from the public health, welfare, mental health and addiction domains. In ten years, schoolBeat aimed to reduce risk behaviours among youth (4–19 years) in the Maastricht region. The projects midterm objectives (2005) focused on establishing sustainable collaboration among schools, health promoting agencies and local authorities. The number and quality of tailored health promotion activities should also be increased in this period. In order to pursue these objectives a systematic plan of coordinated support for tailored school health promotion policy was developed. The plan was based on the principles of intervention mapping [17] and tailored to the possibilities and pitfalls of the educational system and the health system in the Netherlands. Forms of action research were used in combination with literature reviews and expert consultations in writing the plan [18-20]. However, programs cannot be developed based on expertise and authority alone. It requires full participation of all stakeholders [21]. Hence, the development of schoolBeat included participation of stakeholders from the health, welfare, and education sectors and the government. This is a common type of collaboration in school health promotion in the Netherlands [22-25].

The DISC-study was part of the four-year grant for the development and evaluation of the schoolBeat-strategy. The contribution of the stakeholders to the schoolBeat-collaboration itself was not covered by the national grant. This was paid for by the stakeholder-organizations themselves following their tasks and responsibilities in the area of school health promotion.

As it was the first time the DISC-model was used in its current form, exploring its usability for assessing intersectoral collaboration in general was the primary aim of this study. In this paper we will specifically address the differences indicated by DISC-based comparisons between the sectors participating in the schoolBeat-collaboration: schools, support organizations and governments

### The DISC-model

Effective health promotion alliances require management skilled in networking, knowledge-sharing and partnership creation and support [26-28]. Assessment of a potential or existing health promotion collaboration, in addition to needs assessment in the health promotion setting, is required to enable systematic planning of strategies for program development, implementation and maintenance [29,30]. Hence, management skills regarding ongoing evaluation of the collaborative status of the alliance are needed as well [11,14].

The DISC model was developed to systematically support such evaluations. The model goes beyond the more traditional evaluation models used in health promotion, which focus primarily on the implementation and effects of single intervention programs. DISC describes factors affecting the evolution of collaboration. The model focuses on the interaction between project management, collaborating partners as a whole (i.e. collaborative support), project organization and factors in the wider context, and their impact on the subject of the collaborative process. At the level of the 'collaborative support' the model distinguishes between 'perceptions', 'intentions' and 'actions'. Each construct is assessed by a set of indicators. The term 'sustainable' refers to the aim of the collaboration to continue after the initial project phase has ended, without committing themselves to an ever lasting collaboration. If collaborations do not aim for continuation, DISC analysis is not appropriate.

**Figure 1:**
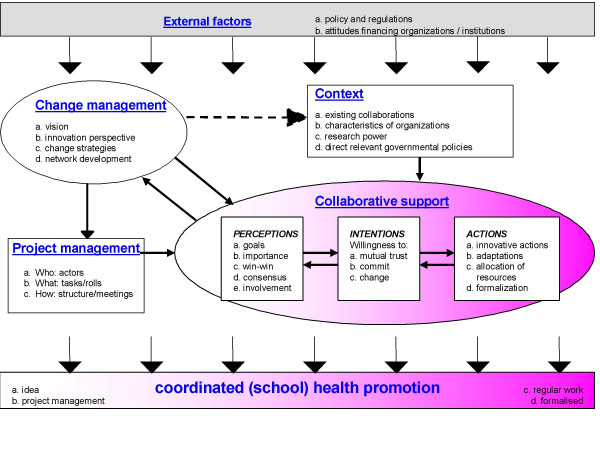
The DIagnosis of Sustainable Collaboration (DISC) model.

Figure 1 presents the DISC-model; the DISC-constructs are delineated in Table 1. Generally, the DISC-model links assessments of the collaborative process directly to the real-life context in which the intervention or set of interventions is developed, implemented and, if successful, maintained. This makes the model appropriate for case study designs as described by Yin and advocated by others [31-33].

**Table 1:** General description of constructs and indicators of the DISC-model

**Construct**	**Scales**	**General description per construct**
**External factors**	1) Policy and regulations 2) Attitudes of financing bodies	*The collaborative process is influenced by a number of factors that are beyond the control or influence of the alliance itself:* 1) Clear, preferably inter-sectoral policies, laws and regulations providing challenging and sound goals for health promotion may enhance the collaborative process. Limiting factors may be diffuse borders between policy domains, contradicting policies of different public sectors and policies focusing on the transformation of public organizations into private enterprises. 2) An encouraging and accommodating attitude of financing bodies and commitment to provide the necessary funding over a longer period to prevent a brain drain from starting during the initial developmental phase, supports the collaborative process.

**Context**	3) Existing alliances 4) Characteristics of organizations 5) Research power 6) Direct relevant governmental policies	*The collaborative process evolves in a context which can be influenced by the partners themselves* When parties have more positive experiences with each other in previous collaborative processes, need less energy for internal changes, have more research power and feel more supported by policies which they can influence as well, they are more open to sustainable collaborative process supporting inter-sectoral health promotion.

**Change Management**	7) Vision 8) Innovation perspective 9) Change strategies 10) Network development	The aspired change requires management by one or a small group of leaders. In order to establish a successful collaboration individual and collective leadership skills are necessary to guide the developmental process. Change management strategies should fit the chosen innovation perspective and be supportive of the health promotion subject. The most relevant actors are included, and where missing, this will be accomplished by extending the network of the leaders of the collaborative process.

**Project – management**	11) actors, task & structure (who, what and how)	*During the development and initial implementation phase the collaborative process is dealt with as a project in a project management structure*. This includes deciding who are the actors in the project, what do they need to do and how do they operate (planning, procedures, evaluation, communication, etcetera). This project management structure fades out when the subject of the collaborative process is (close to) being integrated in regular work and the alliance becomes self-supportive.

**Support** **based on intersectoral collaboration**	**Perceptions**: 12) Goals 13) Importance/Win-win 14) Consensus 15) Involvement	*The collaborative support can be assessed on the levels of perceptions, intentions and actions of the parties involved*. Intersectoral collaboration evolves more smoothly when participating organizations share goals and interests, perceive positive outcomes supportive of their own goals, are able to reach consensus on the goal of the collaborative process and are of the opinion that the most relevant parties are involved in the collaborative process.
	
	**Intentions to**: 16) Mutual trust 18) Commitment 19) Change	Parties involved should start with the intention to trust each other (if not present, this needs to be worked on first), the intention to commit themselves to the collaborative process and its subject and the intention to make changes within ones own organization, if needed, in favor of the collaborative process.
	
	**Actions**: 20) Innovative actions a) Adaptations b) Reallocation of resources 21) Formalizations	The collaborative process may induce a wide variety of actions, varying from the implementation to major innovations within ones own organizations to the inclusion of relatively minor adaptations of regular procedures. The actions may involve a reallocation of resources as well. Whatever actions result from a collaborative process, it is important that these are formalized in order to enhance sustainability. The level of formalization needed depends mainly on the type of action itself.

**Coordinated Health Promotion**	22) From **idea **and **project management **to **formalized regular work**	The collaborative process influences the development of the coordinated (school) health promotion and supports the move towards sustainability (goal): Under continuous influence of the collaborative process, an idea is elaborated and develops into regular working routine being formalize. During this process the subject of the collaborative process evolves: it 'changes color' under influence of the collaborative process itself.

The model is based on a literature review and initial experiences with an explorative research model introduced in the early 1990's. The initial model was used in multiple case studies in collaborative home care services in the Netherlands [34,35]. The model proved to be valuable to case study research in home care and in shared care studies [36]. As no suitable models from the area of health promotion were detected, and experience in the research group with this care-model already existed, the care-model was used as a starting point. It should be noted that the initial model focuses on general elements important to interorganizational collaboration, organizational behavior and planned organizational change [3,11,14,37]. The assumption is that the collaboration puts a strain on the participating organizations and requires them to change in a minor and in specific areas in a major way. This is based on the open systems school of thinking: organizations are social systems that interact with their environment aiming to meet internal needs as well as needs of the environment [38,39].

Ruland and colleagues slightly modified the initial model to be applicable to organizations involved in health promotion alliances, with a strong focus on formalization [40]. However, true formalization of a sustainable alliance is a phase many health promotion alliances may never reach or wish to reach. Like Ruland and colleagues, often reviews and studies on collaborations seem to fail to recognize that the goal of their collaborative process (sustainable coordinated health promotion) may change under influence of the collaborative process itself. Gillies already recommended flexibility in project planning and working procedures, to enable the management of environmental changes at different levels [41]. This can be taken one step further by allowing for adaptations to the project or strategy itself as a result of the collaborative processes, influenced among other things by environmental changes. Hence, the focus of the collaboration may 'change color' during the transition period from idea to formalized activity. This is reflected in color change of the bar at the bottom of the DISC-model. We also added the indicator 'research power' in the context-construct. This was based on the finding that the absence or presence of scientific evaluation influences the level of successful implementation of care innovations in organizations [42].

In this article we describe the application of the model to the initial phase of the schoolBeat collaboration to delineate its possibilities and limitations in enhancing intersectoral collaborative processes in health promotion. The study was conducted in the 2003–2004 period, one year after the initiation of the collaborative process.

## Methods

The study combined quantitative research (a cross-sectional survey), qualitative research (structured interviews) and document analysis. The primary aim of the study was to pilot the DISC-model as an instrument for diagnosing opportunities and impediments of collaborative processes. The practical aim of the pilot for those involved in the alliance diagnosed was to identify differences between groups of stakeholders (i.e. education, health/welfare and government) in school health promotion regarding the DISC-constructs (see Table 1), resulting in recommendations for improving the collaborative process itself.

### Sample

A questionnaire was sent to fifty-five schoolBeat-stakeholders identified by the project management: 19 people from the education domain, 19 people from the support organizations and 17 from municipalities and provincial government. The main criterion for stakeholder selection was a basic understanding of the collaboration studied, expected by the project management. As questionnaires and interviews took place only one year after the start of the schoolBeat-project, no professionals had dropped out at the time of the study. About half of the people from the education domain were active as pupil care coordinators in their schools for secondary education with responsibilities for school health, with the other half fulfilling management positions in primary or secondary educations responsible for preventive pupil care. From the support organizations CEO's, members of the schoolBeat-project group (management level) and schoolBeat advisors were identified as schoolBeat-stakeholders. Professionals participating in the regional youth meeting of public servants or the meeting of aldermen responsible for local and regional youth policy were included in the stakeholder group as well.

Fourteen of the 55 stakeholders were invited to participate in a semi-structured interview. In addition, three influential stakeholders who did not participate in the survey and who were identified by the project management as stakeholders with a presumed negative attitude towards the schoolBeat-alliance, were approached to be interviewed as well. This included an alderman of one of the smaller municipalities, a school director and someone from the support organizations. Of the interview group, four worked in the education sector, five for a support organization and three for a local or regional government.

### Measurement

For the quantitative part, a survey was developed based on a self-evaluation survey for change managers of collaborative processes in primary health care using the original explorative research model [43]. The questionnaire was adjusted to reflect the constructs of the DISC-model and to focus explicitly on the whole-school health collaboration studied. A collaboration expert and a healthy school expert tested face validity of the questionnaire regarding its fit with whole-school health collaboration and the DISC-constructs. The questionnaire was improved on the basis of their suggestions. This was followed by a pre-test among four participants of a youth prevention alliance in a different region. The used instrument is available in Dutch only [see Additional file 1]. The survey consisted of 22 scales operationalizing all the main concepts from the DISC model (see Table 1) with 3 to 8 items. Except for the dichotomous items in the *existing-collaborations *a five point scale was used (1 being 'totally disagree' and 5 being 'totally agree'). Table 2 reports the number of items and the Cronbach's alpha of each scale. The actual instrument is in Dutch (see Appendix).

**Table 2:** Reliability, average and analyses of variance results per scale distinguishing sum-scores between public health, education and public service stakeholder-groups

**DISC-construct**	Number of items	Cronbach's Alpha^1^	Mean (SD)	F
**External factors**				
▪ Policy and regulations	3	0.48	-	-
▪ Attitudes of financing organizations	4	0.74	2.68 (0.89)	0.16

**Context**				
▪ Existing collaborations	3	0.31	-	-
▪ Organizational characteristics	8	0.61	3.71 (0.50)	3.27^0^
▪ Research power	3	0.76	3.32 (0.82)	0.63
▪ Relevant policies	4	0.75	2.95 (0.78)	1.18

**Change management**				
▪ Vision	3	0.79	4.28 (1.00)	0.02
▪ Innovation perspective	6	0.60	3.89 (0.42)	1.13
▪ Change strategies	4	0.67	4.01 (0.55)	10.49**
▪ Network development	4	0.65	4.07 (0.55)	4.94*

**Project management**	5	0.87	3.29 (0.81)	4.69*

**Collaborative support:**				
▪ Perceptions				
▫ *Goals*	9	0.90	4.39 (0.59)	0.63
▫ *Importance/win-win*	4	0.84	3.84 (0.75)	1.23
▫ *Consensus*	5	0.82	3.82 (0.72)	0.27
▫ *Involvement*	5	0.64	3.83 (0.61)	1.41
▪ Intentions				
▫ *Willingness to trust*	3	0.69	4.10 (0.62)	0.29
▫ *Willingness to commit*	4	0.69	3.31 (0.74)	4.47*
▫ *Willingness to change*	4	0.52	-	-
▪ Actions				
▫ *innovative actions & adaptations*	5	0.67	2.86 (0.79)	3.98*
▫ *resources*	2	0.40	-	-
▫ *formalization*	2	0.33	-	-

**Coordinated School Health Promotion**	8	0.90	3.53 (0.66)	1.27

** = p < 0.01; * = p < 0.05; ^0 ^= p < 0.10

^1 ^For scales with two items, Pearson's correlation coefficient is presented.

The interviews were semi-structured to address all constructs of the DISC-model and lasted one hour on average, covering all 22 scales. Where survey participants had presented the researchers with unexpected or unclear answers in the questionnaires, this was addressed in more detail in the interview. After asking the participants for permission, all interviews were taped and written down. Because there were only twelve participants involved, the interviews were analyzed manually. The transcriptions of the interviews were categorized in the clusters of the DISC-mode and quotations were labeled due to the cluster it referred to. Matching quotations on the same questions were counted and finally all quotations of the participants were summarized and described by each cluster. Additionally, documents such as minutes of meetings, project descriptions and correspondence with schools and local and regional governments were analyzed based on the DISC-model as well.

### Statistical analyses

Cronbach's alpha was calculated for the scales operationalizing the distinct DISC-constructs (Table 2). For scales with only two items Pearson Correlation was calculated. We accepted a reliability score of 0.60 or above because of the explorative nature of the case study and the relative low number of possible participants in the survey.

Analysis of variance was conducted to test for differences on the DISC-constructs between stakeholders from the education sector, the public sector and the support sector. Where differences among the means were significant (p < 0.05) or a trend was observed (p < 0.10), the Bonferroni test was used to test differences between groups. The transcribed interviews and retrieved documents were scored manually according to the definitions of the DISC-constructs (Table 1).

### Procedure

The survey was sent out to 55 schoolBeat-stakeholders in May 2003, with a 4-week deadline. A letter providing information on the aim of the study and confidentiality of individual answers accompanied the survey to fulfill informed consent requirements. The same information was provided with the invitation for the interviews. Semi-structured interviews were conducted during autumn 2003, following up on results and questions risen from the document analysis and the first analysis of the survey-results. Approval by an ethical committee was not required for this non-medical study.

## Results

Of the 55 stakeholders invited to fill in the DISC-survey, 17 out of 19 people responded from the education sector (90% response), 17 out of 19 from the support organizations (90% response) and 8 out of 17 from the local and regional government (47% response). Overall response amounted to 76%. For the interviews, 12 persons of the 14 approached agreed to be interviewed.

Reliability analyses of the survey revealed that five scales were not sufficiently reliable: the policy and regulations scale, the existing-collaborations scale, the willingness to change scale, the resources scale and the formalization scale. These scales and their items were not included in further analyses. The reliability coefficients, averages per construct and the results of the analyses of variance per construct are presented in Table 2.

Table 2 shows relative low scores (< 3) for the perceived attitudes of financing organization scale (external factors-construct), relevant policies (context-construct) and innovative actions and adaptations (actions construct). The innovative actions and adaptations scale showed significant differences between the groups, with education stakeholders perceiving most innovative actions and adaptations (Mean 3.33; SD 0.37) and public service stakeholders perceiving least innovative actions and adaptations (Mean 2.11; SD 0.54).

Relative high scores (> 4) were found for three of the four scales in the change management construct with the change strategies and network development scales indicating significant differences between the groups (see Table 3). 'Goals' and 'willingness to trust' yielded also relative high scores without significant differences between groups.

**Table 3:** Significantly differing group averages regarding DISC-constructs

**DISC-construct**	Education (n = 17) mean (SD)	Health Promotion (n = 17) mean (SD)	Public Service (n = 8) Mean (SD)
**Context**			
▪ Organizational characteristics:	3.92 (0.49)^a^	3.68 (0.46)	3.40 (0.45)^b^
▫ *Open to innovation*	*4.12 (0.86)*	*4.47 (0.62)*^*a*^	*3.50 (0.93)*^*b*^
▫ *Fully staffed (no long-standing vacancies)*	*4.53 (0.62)*^*a*^	*4.29 (0.85)*^*a*^	*3.43 (0.73)*^*b*^
▫ *Major organization change processes*	*4.47 (0.80)*^*a*^	*4.24 (0.83)*	*3.38 (1.30)*^*b*^
▫ *Financial problems now or expected in the near future*	*4.19 (1.24)*	*3.69 (0.85)*^*a*^	*4.75 (0.46)*^*b*^

**Change Management**			
▪ Change strategies	3.72 (0.39)^a^	4.43 (0.32)^b^	4.01 (0.75)
▪ Network development	3.99 (0.44)	4.34 (0.39)^a^	3.69 (0.80)^b^

**Project Management**	2.91 (0.78)^a^	3.70 (0.67)^b^	3.26 (0.81)

**Collaborative Support**			
▪ Intentions: willingness to commit	3.19 (0.52)	3.67 (0.86)^a^	2.83 (0.60)^b^
▪ Actions: Innovative actions & adaptations	3.33 (0.37)^a^	2.83 (0.77)	2.11 (0.54)^b^

Means with different superscripts are statistically different (p < 0.05)

Table 3 presents the constructs that revealed at least one significant difference between respondents of the three sectors: education, health and public service. Only for the first construct – organizational characteristics – differences found at item level are shown to illustrate the added value of further exploration in a DISC-study. The analyses revealed that stakeholders from the public sector indicated to be less committed to the schoolBeat initiative and to have incorporated fewer changes supporting whole-school health promotion compared to their school and support partners. Additionally, they reported to be least open to innovation and to experience the most financial problems now or in the near future. Health promotion staff reported the highest level of intention to commitment while education staff reported the highest level of innovative actions and adaptations. Participants from the education domain reported to be better staffed but also to experience more major organizational changes compared to the public service. The health promotion support staff involved experienced higher level of change strategies and project management compared to the education staff.

In this explorative phase, the interviews seemed to shed more light on specific opportunities and impediments of the collaboration studied. Project-related documentation, including goals and a project management structure, was combined with to the survey-results in preparation for the interviews. During the interviews it became apparent that stakeholders from the three sectors used different interpretations of the goals of the schoolBeat collaborative. Differences were related to the health promotion versus pupil care debate (i.e. *'Why talk about prevention when we have not enough support for our individual pupil care problems?'*). From the education-stakeholders we learned that they were skeptical about yet another new approach, with mixed evaluations of previous attempts. Nonetheless, these education-stakeholders saw opportunities in linking the schoolBeat approach to a safe-school approach already operating in schools. They seemed keen on improvements in the whole-school pupil care continuum with policies changing towards *full inclusive *education. This means that children with special needs should be able to attend regular schools, like in the US and other Anglo-Saxon countries. This was somewhat contrary to what health promotion stakeholders' wished to achieve with the schoolBeat-approach focusing primarily for collaborative improvements in the health promotion end of the integral pupil care continuum.

Regarding the DISC-constructs, the interviews and document analysis provided additional insights. For example, those interviewed found it difficult to distinguish innovative actions from adaptations within the 'action'-construct. The results of the interviews and the document analyses regarding the schoolBeat-alliance combined are summarized per DISC-construct in Table 4.

**Table 4:** Results of the DISC-analyses based on the transcribed interviews and document analysis

	**Education**	**Health Promotion**	**Government**
*External factors*	***Policies and regulations*** Inclusive education: this puts a strain on individual pupil care in regular schools inhibiting attention for health promotion and prevention at group level ***Attitudes financing bodies*** Lump sum financing for schools Additional finances for pupil care in schools in deprived areas	***Policies and regulations*** - ***Attitudes financing bodies*** - Introduction of free market mechanisms in health promotion and welfare feeds feelings of competition among partners, especially at the advisory level	***Policies and regulations*** - Decentralization of tasks and regulations from national to regional and local governments

*Context*	***Existing collaborations*** - A collaborative history with youth health care and youth monitoring with limited results so far - For schools in deprived areas a collaborative history with youth welfare with mixed evaluations - Safe schools working group with municipality, police, justice and public health ***Organizational characteristics*** - Autonomy of teaching staff - No planning and reporting of HP actions ***Research power*** - Not present locally ***Relevant local policies*** - Safe schools policy - Youth welfare in schools	***Existing collaborations*** - The public health institute has positive collaborative experiences with all HP partners. - Limited collaborative experiences exists among the partners themselves - Participation in safe schools working group ***Organizational characteristics*** - Innovation minded management - Limited internal support for HP ***Research power*** - Around half of the HP organizations involved have academic research experience. ***Relevant local policies*** - Youth welfare in schools as pilot welcomed by schools - Regional shared care networks for youth policy	***Existing collaborations*** - Negative experiences prevail with the institute providing most of the schoolBeat-leadership. - Positive attitude towards another schoolBeat-partner - Neutral towards other parties involved. - Coordinator of the safe schools working group ***Organizational characteristics*** - Influenced by political changes - Bureaucratic ***Research power*** - A lack of expertise and direct interest in generating and using academic evidence. ***Relevant local policies*** - Regional shared care networks for youth policy

*Change Management*	**Innovation perspective** - Based on educational expertise **Change strategies** - Not actively applied **Network development** - Inclusion of leadership of a primary schools' representative and a care coordinator would be desirable	**Innovation perspective** - Based on empirical evidence combined with existing expertise among partners **Change strategies** - Network meetings - Education of HP staff **Network development** - More active support from HP management is desired.	**Innovation perspective** - not clearly defined **Change strategies** - open for information regarding the collaborative process **Network development** - wish to start sharing information with members of local and regional politics

*Project management*	No participation of schools in the project organization at this stage.	**Whom: actors** filled in by the HP organizations only **What & How: tasks & structure** described by the coordinator and agreed upon by the management of partner organizations	No official governmental participation One civil servant participated in the project group but started as education support staff before changing jobs and was allowed by his new employer to keep participating once joining an education department at municipality level.

*Support* Perceptions Intentions Actions	**Perceptions**: *goals/importance* - Quality improvement - Creation of a pupil care support continuum **Perceptions**: *win-win* - Workload sharing regarding pupil care with organizations outside the school - School health profiles add to internal school assessments for planning purposes. **Perceptions**: *consensus* - Tailored support from a single point of contact - Unease regarding the attention not yet paid to individual pupil care **Perceptions**: *involvement* - Direct involvement of public service is missed by some **Intentions**: *willingness to trust* - Seems present based on previous experiences with the HP partners **Intentions**: *willingness to commit* - Based on perceived added value most school administrators are willing to commit **Actions**: *innovative actions and adaptations* - Appointment of prevention teams in the first schools - High level of participation in evaluation **Overall: sufficient**	**Perceptions**:*goals/importance* - Quality improvement - Strengthening HP within schools - Creation of a HP support continuum **Perceptions**: *win-win* - Workload sharing provides a win to all HP organizations involved **Perceptions**: *consensus* - Consensus is present regarding the basic outline of the methodology - Tension is present regarding specific elements of the methodology **Perceptions**: *involvement* - Direct involvement of schools and public service is missed by some **Intentions**: *willingness to trust* - Feelings of competition among HP advisors and managers **Intentions**: *willingness to commit* - Moderate to high, with major differences among organizations **Intentions**: *willingness to change* - Is present, but partners are keen on experiencing some positive results first and do not know yet what exact changes would be necessary **Actions**: *innovative actions and adaptations* - Appointment of schoolBeat – support staff to schools by the four key-partners - Description of support options in unified format by all partners **Overall: sufficient/good**	**Perceptions**: *goals/importance* - Improvement of efficiency and quality of HP and pupil care support - Deleting overlap in HP support **Perceptions**: *win-win* - Unclear about the value for the municipalities involved **Intentions**: *willingness to trust* - Benefit of the doubt based on the core ideas of the collaborative subject: whole-school health promotion **Intentions**: *willingness to commit* - Moderate, as long as requirements set at the start are met **Actions**: *innovative actions and adaptations* - Nearly absent - Limited participation in evaluation **Actions**: *resources* - The collaborative process needs to produce a methodology which entails no additional costs to local governments **Overall: sufficient/good**

*Coordinated school health promotion*	Idea – start of a project	Main focus on project (beyond idea phase)	Main focus on project (beyond idea phase)

Competitive feelings of one of the initial schoolBeat partners surfaced during the DISC-pilot. These feelings were a result of the decision by the municipalities to transfer their powers regarding the financing of certain types of school support to the schools (see Table 4). The employees of the partner providing the support involved, seemed to feel threatened by this decision. To those involved, it was not sure whether this was a temporary phenomenon that could be overcome with trust among the partners or that it would be a lasting complicating factor with possible destructive effects on the schoolBeat-alliance. Therefore, it was recommended to pay special attention to developments leading to a re-introduction of competition elements between collaborating partners. The competitive feelings should be recognized by all partners and discussed in the perspective of dealing with a complicating factor caused by external factors.

The interviews further suggest that education professionals were least positive about the schoolBeat-collaboration. Here it should be known that the preparation of the schoolBeat-alliance started in 2001 with representatives of support organizations and local communities. Discussions with the education sector started in 2002 at management level, followed by an introduction of the schoolBeat methodology late 2002, spring 2003 at the school level. Misconception regarding the schoolBeat-goals of the education stakeholders complicated this delayed start even more. As the education stakeholders were expecting an improvement on the whole pupil care continuum due to schoolBeat, they were in for a deception with the schoolBeat-methodology focusing on school health promotion and prevention only. Hence, this provides room for improvement when the schoolBeat-alliance continues to involve education-representatives and to work towards shared goals.

A summary of the combined results of the DISC-pilot, with recommendations for improvement of the collaborative schoolBeat-related processes was forwarded to the schoolBeat project team. The recommendations included extending the project management over a longer period of time; strengthening communication with policy-makers; providing a communication boost regarding the proposed methodology and the extra financial possibilities targeting stakeholders' colleagues; further development and clarification of the shared methodology itself; and intensifying information sharing among the key-stakeholders. Although not all recommendations could be carried out straight away due to a variety of constraints, they were all accepted as intermediate goals for the alliance by the schoolBeat-partners.

## Discussion

In this study we took up the challenge to learn from previous health-related collaborations [10,35,44], and the diagnostic models used. Based on a literature review and practical experiences, we reshaped and extended the WIZDIZ-model into the DISC-model: diagnosis of sustainable collaboration. We explored the use of the DISC-model at an early stage in a collaborative and incremental process developing a comprehensive and tailored strategy for whole-school health promotion. Our aim was to provide a description of the current state of affairs regarding the collaboration in order to enable the selection and implementation of suitable change strategies.

The DISC-analysis provided us with a cross-sectional picture of a complex phenomenon: an intersectoral health promotion alliance including health/welfare, education and government. We found that involving stakeholders from the three groups involved as well as using multiple data sources complemented the picture created. It seemed to increase validity of the findings.

The DISC-model provides a comprehensive overview of factors involved in inter-agency collaborations. Especially the qualitative strand of the study seemed to provide the most specific insights into the current status of the collaboration. This should not be limited to document analyses only as this may not provide insight into disquiet among partners or possible other – not formally reported – negative aspects of the current status of a collaborative process [8,45,46]. For example, in the DISC-survey 'research power' did not reveal significant differences between groups based on survey-data, where document analysis did manage to provide useful information on the differences between stakeholder groups regarding this construct (see Table 4).

Our study clearly supports the value of using a systematic approach to monitoring the state of the art of interorganizational collaboration. Up-to-date information regarding windows of opportunity as well as impediments for collaborative change revealed by DISC-monitoring enhanced the selection of suitable strategies for collaborative problem solving.

To enable the use of DISC-based monitoring in other alliances that lack support of a professional research team, a short DISC-checklist for the project management would be useful similar to the previous developed for the WIZDIZ-model [47]. As the added value seemed to come from including different perspectives on the collaborative process, project managers using such a checklist should be encouraged to ensure representative stakeholder-input. Especially stakeholders from domains least familiar to the project manager may provide the most useful input for collaborative change.

To test the DISC-model itself (for example by using structural equation modeling), more participants would need to be included. As the model focuses on collaborative processes, only those people can be asked to participate who are considered to be stakeholders in these processes. This limits the number of possible participants in such a study. To overcome this problem, it could be worthwhile to apply a more generalized survey to comparable alliances simultaneously. In our case it would be preferable to stay with whole-school health alliances in order to be able to work with comparable sets of stakeholders. Another option is turning the DISC-analyses into a longitudinal study, as suggested by Feinberg and colleagues regarding clarification of causal direction in network analysis [48]. As the collaborative process evolves over time, we expect DISC-analysis to reveal a flow in the model from the idea phase toward organizational routine as the collaborative process matures. This is in line with the ideas of Plsek and colleagues regarding complexity science in which they advocate treating organizations as complex, adaptive systems [49,50]. Nonetheless, we strongly believe that successful DISC-based analyses should never be conducted using a quantitative survey only.

A weak aspect of the DISC-survey was the assessment of the existing collaborations construct and the formalization construct. Available literature in health promotion is unclear on these topics [48,51,52]. For example is it closeness or number of relationships within the network that counts? And: what needs to be formalized as a requirement for sustainability? Or, what do participants actually mean when they talk about sustainability? A recent study by St. Leger indicated a wide variety of definitions of sustainability among participants in the same collaborations [52]. Further research in the area of sustainability is required.

The DISC-model does not state how to advance and improve the collaborative process. Based on the systematically gathered evidence, informed decisions are possible for further action by those involved [17,45]. In particular, those who are supposed to fulfill leadership tasks should be aware of the different DISC-constructs and current DISC-status of the alliance in order to do so. In our pilot, a literature search was conducted parallel to the DISC-analyses in order to provide evidence-based recommendations. For example the recommendation to extend the project management over a longer period of time was suggested by some of the interview participants but was also supported by recommendations of a support structure in current health promotion literature [7,53]. Additionally, as pointed out by Nutbeam policy makers do not make use of scientific evidence regularly [54]. Hence, it comes as no surprise that the DISC-analysis indicated that the 'research power' of the government is rather limited and that better communication with politicians is to be recommended here.

Tuckman's four-stage model of group development processes [In: [45]] – forming, storming, norming and performing – could add to the understanding of collaborative processes in health promotion. In the studied case, staff involved in the alliance from education, health promotion and government appeared to be in different developmental phases. The health promotion partners seemed to be in the storming phase in which some unease and conflict was present amongst each other. The education and governmental stakeholders were still in the forming phase of orientation and getting acquainted. Awareness of this aspect could help the leadership to prepare for the storming phase among these education and governmental stakeholders once they had moved through the initial getting-to-know-each-other-better phase. For the health promotion partners, time and effort needed to be spent on consensus seeking in order to advance their input in the collaborative process. Therefore, clarification of the developed methodology was one of the evidence-based recommendations as well.

Based on the first use of the DISC-model and additional literature searches, we modified the following elements of the model:

- the 'change-management' construct was changed into 'leadership' incorporating both individual leadership as well as collective leadership [26,37,55]. According to Weiss and colleagues this enables bridging diverse cultures and boundary-spanning functions as well as revealing and challenging assumptions that limit thinking and action [51].

- Being a continuum, the concepts of 'innovative actions' and 'adaptation' were combined into the concept 'changes' within the 'action'-construct.

- The subject of the collaboration (represented by the bottom bar of the model) was simplified into a continuum starting as an 'idea' and leading to a 'routine' in its most pure form [33,56]. This eliminates the term 'formalized' in the subject of the collaboration, being already included in the 'collaborative support – action' construct.

- Additionally, based on the stakeholder theory, which includes the community's notion of social responsibility, and the institutional theory [57], we introduce the concept of 'society values' as part of the external factors in the DISC-model.

## Conclusion

The DISC-model is more than just the sum of the different parameters provided in the literature on interorganizational collaboration, organization change, networking and setting-approaches such as trust, relationships between partners and interpersonal connections, project management (including identification of roles and responsibilities), leadership, flexibility in working practices, institutionalization [3,5,26,37,41,45]. DISC-analysis provides indications regarding the links between these parameters and – potentially – enabling the detection of change in the combined collaborative parameters over time.

Linking a simplified DISC-analysis to the evaluation of single interventions in (school) health promotion based on a collaborative effort, may add to the explanation of the results of such an evaluation study. Context assessments have been advocated in several recent (school) health promotion studies [8,46,58]. The DISC-analysis provides insight into the organizational context of the intervention and indications for the sustainability of such an intervention as well as indications for the transferability of the evidence provided. This is almost all about the organizations who will have to support and implement the intervention structurally. Hence, DISC-analysis could help preventing type III errors from occurring in effectiveness studies: a health promotion intervention supposedly proves to be ineffective when it is actually the management and implementation which fails [59].

With collaborative processes inevitably linked to health promotion, thorough analysis of these processes should be part of any participatory action research approach to enhance health promotion via intersectoral collaboration. The DISC-analysis model offers a promising comprehensive evaluation framework looking at the status of the collaborative process and its impact on the goals of the health promotion initiative. Further exploration of the proposed DISC-constructs is warranted as well as simplifications of its use.

## Abbreviations

DISC: DIagnosis of Sustainable Collaboration.

## Competing interests

The authors declare that they have no competing interests.

## Authors' contributions

MTWL developed the DISC-model, conducted the survey-part of the study and drafted the manuscript. IMMV participated in the development of the DISC-model, contributed to the design of the study and helped draft the manuscript. RvdS conducted the interviews, participated in data analysis and in the revision of the article. HPS participated in the design of the study and helped to draft the manuscript. NKV contributed to the interpretations of data and revised the manuscript critically for important intellectual content. All authors read and approved the final manuscript.

## Pre-publication history

The pre-publication history for this paper can be accessed here:



## Supplementary Material

Additional file 1 **The Dutch DISC-questionnaire.** The Dutch DISC-questionnaire as used in the 2003 validation study. Click here for file
