# Supplementary material for: Diagnosis of sustainable collaboration in health promotion – a case study
Source: BMC Public Health. 2008 Nov 7;8:382. doi: 10.1186/1471-2458-8-382 (PMC2605462; doi:10.1186/1471-2458-8-382)
Supplement: Additional file 1 — The Dutch DISC-questionnaire. The Dutch DISC-questionnaire as used in the 2003 validation study. [file 1471-2458-8-382-S1.doc]

# ONDERZOEK

SchoolSlag-samenwerking gericht

op de ontwikkeling van

gecoördineerd en vraaggestuurd integraal schoolgezondheidsbeleid

SchoolSlag staat voor de ommezwaai van aanbodgericht naar gecoördineerde, vraaggestuurde ondersteuning op het terrein van gezondheidsbevordering en preventie in, met en rondom het onderwijs. Met gezondheid bedoelen we hier: fysiek, sociaal én mentaal welbevinden.

Scholen stellen zelf hun eigen prioriteiten op het terrein van gezondheidsbevordering & preventie vast op basis van de eigen subjectieve behoefte gekoppeld aan objectieve cijfers over de eigen schoolpopulatie. Vanuit schoolSlag wordt dit gefaciliteerd door het samenstellen van schoolgezondheidsprofielen en het begeleiden van het verhelderen van de subjectieve behoefte bij medewerkers, ouders en leerlingen.

Scholen kunnen via hun eigen schoolSlag-contactpersoon advies en ondersteuning ontvangen bij het matchen van de beste mogelijkheden (inclusief eigen ideeën en oplossingen) om invulling te geven aan de zelf gestelde prioriteiten op het terrein van gezondheid, welzijn en veiligheid. Dit binnen de randvoorwaarden en uitgangspunten van de eigen school. SchoolSlag heeft zoveel mogelijk beschikbaar aanbod en ondersteuning geïnventariseerd en een visie op kwaliteit van aanbod en ondersteuning ontwikkeld. Tevens legt schoolSlag een koppeling met activiteiten en interventies buiten de school (o.a. Gezondheidswijzer en buurtactivit eiten) zodat deze het eigen schoolgezondheidsplan kunnen versterken. Het Gezonde School Model wordt in schoolSlag gehanteerd als leidraad in de advisering over de door de school gestelde prioriteiten.

De schoolSlag-werkwijze geeft concreet invulling aan vraaggestuurd preventief jeugdbeleid in, met en rondom de setting onderwijs. Gezondheid, welzijn en veiligheid maken hier integraal deel van uit.


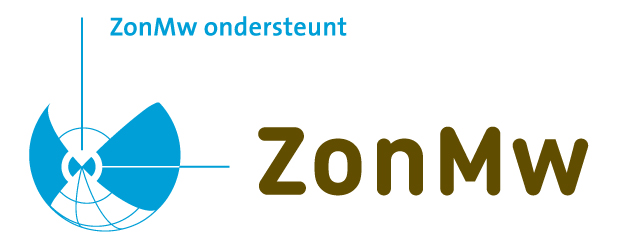


| Het Gezonde School Model  | **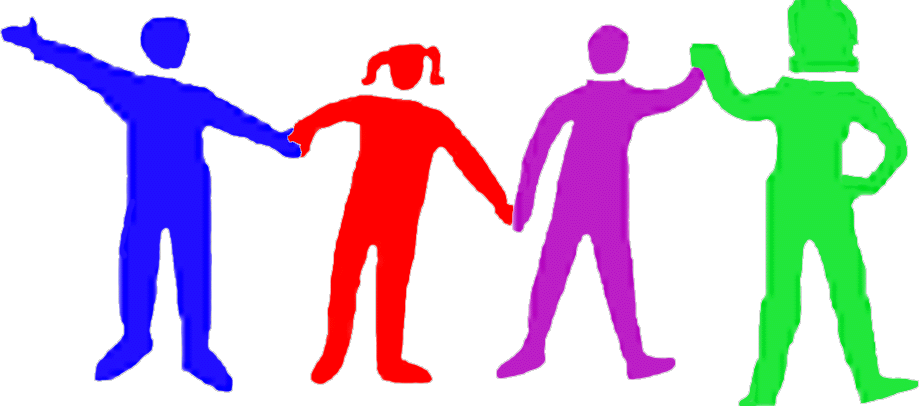** | | --- | |
| --- | --- |

**
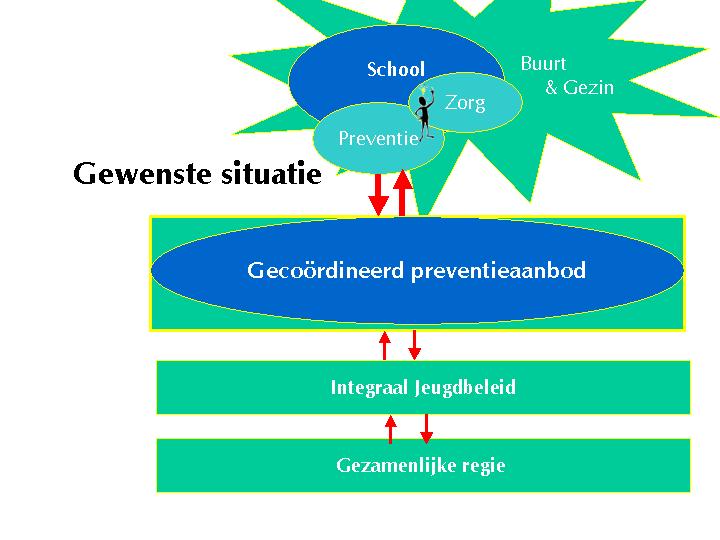
**

# Alvorens te starten

# INTRODUCTIE

SchoolSlag richt zich op de ontwikkeling, implementatie en evaluatie van een innovatieve werkwijze om te komen tot gecoördineerd, vraaggestuurd integraal schoolgezondheidsbeleid in de regio Maastricht-Mergelland. Via de schoolSlag-werkwijze geven regionale instellingen invulling aan een één-loket-gedachte op schoolniveau voor gezondheid, welzijn en veiligheid. Deze ontwikkeling geldt voor zowel basis- als voortgezet onderwijs.

Samenwerking tussen organisaties speelt hierin een belangrijke rol. In deze vragenlijst staat de schoolSlag-samenwerking en de schoolSlag-ontwikkeling. Deze vragenlijst geeft een impressie van de huidige fase waarin de schoolSlag-ontwikkeling en samenwerking verkeert. Het is bovenal een moment-opname, die in 2004 en 2005 herhaalt zal worden.

Omdat deze vragenlijst deel uitmaakt van de evaluatie van schoolSlag, hoop ik van harte dat u de vragen zo waarheidsgetrouw invult. Ook als dit betekent dat uw antwoord negatief of kritisch is. Dit kan inzicht geven in knelpunten in de huidige samenwerking / ontwikkeling. Juist daar kunnen we gezamenlijk van leren en mogelijk ook op verbeteren. Het is belangrijk om de vragenlijst in te vullen zonder verder overleg of raadplegen van documenten. **Uw eigen beleving en interpretatie** van de werkelijkheid is namelijk het belangrijkste uitgangspunt.

Omdat de antwoorden direct gekoppeld zijn aan u en uw organisatie, is het verzoek om deze vragenlijst **niet** anoniem in te vullen. Juist omdat er metingen op andere momenten volgen, is het belangrijk nu al de juiste gegevens van de juiste persoon en organisatie te kunnen koppelen. Gegevens worden vertrouwelijk behandeld.

Invullen doet u door een kruisje te plaatsen bij het juiste antwoord. Als u een antwoord wilt veranderen, maak dan het foute hokje geheel zwart/blauw en zet een kruisje in het juiste hokje. Gemiddeld neemt het invullen van de vragenlijst 30-45 minuten in beslag.

Alvast hartelijk dank!

#### Mariken Leurs

onderzoeker schoolSlag

nb. De vragenlijst maakt tevens deel uit van de wetenschappelijke evaluatie van schoolSlag door GGD ZZL en Universiteit Maastricht. In de regio Westelijke Mijnstreek wordt de vragenlijst ingezet als pre-test.

Dit gedeelte bevat een aantal vragen over externe factoren die van invloed zijn op vraaggestuurd preventief jeugdbeleid, maar niet direct door het RPPJ beïnvloed kunnen worden.

Geef aan of de volgende zaken op het terrein van overheidsbeleid volgens u bevorderend, neutraal, of belemmerend werken voor de samenwerking ten behoeve van vraaggestuurd preventief jeugdbeleid.

| Bevorderend Neutraal Belemmerend Onbekend | | | | | |
| --- | --- | --- | --- | --- | --- |
| **1.1** | De Wet Collectieve Preventie Volksgezondheid (WCPV) | 🞏 | 🞏 | 🞏 | 🞏 |
| **1.2** | Het Nationaal Contract Openbare Gezondheidszorg | 🞏 | 🞏 | 🞏 | 🞏 |
| **1.3** | De wet op de Jeugdzorg | 🞏 | 🞏 | 🞏 | 🞏 |
| **1.4** | Recente landelijke rapporten (RIVM, Raad voor de Volksgezondheid en Zorg, etc) | 🞏 | 🞏 | 🞏 | 🞏 |
| **1.5** | Het huidige onderwijsbeleid | 🞏 | 🞏 | 🞏 | 🞏 |
| **1.6** | Het nieuwe dualisme in de gemeentelijke politiek | 🞏 | 🞏 | 🞏 | 🞏 |
| **1.7** | Decentralisering van taken op het terrein van zorg en welzijn naar de lokale overheid | 🞏 | 🞏 | 🞏 | 🞏 |

| Geef van de volgende stellingen aan in hoeverre u het er mee eens of oneens bent.. Als u over een item géén mening heeft, vult u niets in. | | | | | | |
| --- | --- | --- | --- | --- | --- | --- |
| **.** | | geheel oneens | Neutraal | | | geheel eens |
| **2.1** | De schoolSlag-werkwijze past binnen de huidige prioriteiten van het **lokale** overheidsbeleid. | 🞏 | 🞏 | 🞏 | 🞏 | 🞏 |
| **2.2** | De schoolSlag-werkwijze past binnen de huidige prioriteiten van het **provinciale** overheidsbeleid. | 🞏 | 🞏 | 🞏 | 🞏 | 🞏 |
| **2.3** | De schoolSlag-werkwijze past binnen de huidige prioriteiten van het **landelijke** overheidsbeleid. | 🞏 | 🞏 | 🞏 | 🞏 | 🞏 |
| **2.4** | De schoolSlag-werkwijze past binnen de prioriteiten van mijn eigen beroepsgroep / koepelorganisatie. | 🞏 | 🞏 | 🞏 | 🞏 | 🞏 |
| **2.5** | De schoolSlag-werkwijze is goed inpasbaar binnen de geldende financieringsregels. | 🞏 | 🞏 | 🞏 | 🞏 | 🞏 |
| **2.6** | De doelen van schoolSlag worden maatschappelijk hoog gewaardeerd. | 🞏 | 🞏 | 🞏 | 🞏 | 🞏 |

De financiering van werkzaamheden ten behoeve van gecoördineerde, vraaggestuurde ondersteuning van het onderwijs op het terrein van gezondheidsbevordering & preventie staat centraal in de volgende vragen.

|  |  | Landelijke l  Gemeente Provincie overheid anders | | | |
| --- | --- | --- | --- | --- | --- |
| **3.1** | Wie is voor uw organisatie de belangrijkste financier van werkzaamheden in het kader van vraaggestuurd preventief jeugdbeleid? | 🞏 | 🞏 | 🞏 | 🞏 |
| Indien anders, graag belangrijkste financier invullen:…………………….. | | | | | |
| Geef aan in hoeverre de volgende zaken volgens u van toepassing zijn **(kolom A)** én geef vervolgens aan in hoeverre de opstelling van de financier bevorderend of belemmerd in voor vraaggestuurd preventief jeugdbeleid **(kolom B)**.   |  |  |  | | Ja **A** Nee  1 2 3 4 5 | bevorderend **B** belemmerend  1 2 3 4 5 | | | --- | --- | --- | --- | --- | --- | --- | | **4.1** | De belangrijkste financier van mijn organisatie denkt mee over de ontwikkeling van schoolSlag. | |  | 🞏 🞏 🞏 🞏 🞏 |  | 🞏 🞏 🞏 🞏 🞏 | | **4.2** | De belangrijkste financier interpreteert de geldende financieringsregels ruimhartig. |  | | 🞏 🞏 🞏 🞏 🞏 |  | 🞏 🞏 🞏 🞏 🞏 | | **4.3** | De belangrijkste financier is op uitvoerend niveau direct betrokken bij schoolSlag. |  | | 🞏 🞏 🞏 🞏 🞏 |  | 🞏 🞏 🞏 🞏 🞏 | | **4.4** | De belangrijkste financier is op beleidsmatig niveau direct betrokken bij schoolSlag. |  | | 🞏 🞏 🞏 🞏 🞏 |  | 🞏 🞏 🞏 🞏 🞏 | | **4.5** | Wat kan de belangrijkste financier volgens u doen  om schoolSlag te bevorderen?  🞏 niets  🞏 iets, namelijk: | | | | | | | | | | | |

Via de vragen in dit blok willen we een beeld krijgen van de situatie waarin uw organisatie momenteel verkeert. De genoemde factoren kunnen mogelijk het dagelijkse werk en de schoolSlag-samenwerking beïnvloeden.

Geef aan in hoeverre de volgende zaken volgens u van toepassing zijn op uw eigen organisatie **(kolom C)** én geef vervolgens aan in hoeverre de situatie in uw organisatie de samenwerking met/binnen vraaggestuurd preventief jeugdbeleid bevordert of belemmert **(kolom D)**.

|  |  |  | **Ja**  **C** **Nee**  1 2 3 4 5 |  | **bevordert**  **D** **belemmert**  1 2 3 4 5 |
| --- | --- | --- | --- | --- | --- |
| **5.1** | In mijn organisatie zijn ingrijpende veranderingsprocessen aanwezig. |  | 🞏 🞏 🞏 🞏 🞏 |  | 🞏 🞏 🞏 🞏 🞏 |
| **5.2** | Mijn organisatie heeft huisvestingsproblemen. |  | 🞏 🞏 🞏 🞏 🞏 |  | 🞏 🞏 🞏 🞏 🞏 |
| **5.3** | Mijn organisatie kent een laag personeelsverloop. |  | 🞏 🞏 🞏 🞏 🞏 |  | 🞏 🞏 🞏 🞏 🞏 |
| **5.4** | Mijn organisatie heeft een volledige personele bezetting. |  | 🞏 🞏 🞏 🞏 🞏 |  | 🞏 🞏 🞏 🞏 🞏 |
| **5.5** | De beslislijnen in mijn organisatie zijn kort. |  | 🞏 🞏 🞏 🞏 🞏 |  | 🞏 🞏 🞏 🞏 🞏 |
| **5.6** | Mijn organisatie kampt met financiële tekorten. |  | 🞏 🞏 🞏 🞏 🞏 |  | 🞏 🞏 🞏 🞏 🞏 |
| **5.7** | Mijn organisatie staat open voor vernieuwingen. |  | 🞏 🞏 🞏 🞏 🞏 |  | 🞏 🞏 🞏 🞏 🞏 |
| **5.8** | Orde en systematisch werken gebaseerd op vastgestelde procedures en bevoegden zijn kenmerkend voor mijn organisatie. |  | 🞏 🞏 🞏 🞏 🞏 |  | 🞏 🞏 🞏 🞏 🞏 |
| **5.9** | Bij besluitvorming in mijn organisatie zijn vertrouwen en wederzijds respect belangrijker dan de doelen waar mijn organisatie voor gaat. |  | 🞏 🞏 🞏 🞏 🞏 |  | 🞏 🞏 🞏 🞏 🞏 |
| **5.10** | Mijn organisatie hecht veel belang aan evaluatieonderzoek. |  | 🞏 🞏 🞏 🞏 🞏 |  | 🞏 🞏 🞏 🞏 🞏 |
| **5.11** | In mijn organisatie is/zijn expert(s) op het terrein van evaluatieonderzoek aanwezig die tijd hebben om mij te adviseren. |  | 🞏 🞏 🞏 🞏 🞏 |  | 🞏 🞏 🞏 🞏 🞏 |
| **5.12** | Mijn organisatie maakt regelmatig gebruik van externe onderzoeksorganisaties. |  | 🞏 🞏 🞏 🞏 🞏 |  | 🞏 🞏 🞏 🞏 🞏 |
| **5.13** | Mijn organisatie heeft veel invloed op het onderwijsbeleid in de regio. |  | 🞏 🞏 🞏 🞏 🞏 |  | 🞏 🞏 🞏 🞏 🞏 |
| **5.14** | Mijn organisatie heeft veel invloed op het gezondheidsbeleid in de regio. |  | 🞏 🞏 🞏 🞏 🞏 |  | 🞏 🞏 🞏 🞏 🞏 |
| **5.15** | Mijn organisatie heeft veel invloed op het welzijnsbeleid in de regio. |  | 🞏 🞏 🞏 🞏 🞏 |  | 🞏 🞏 🞏 🞏 🞏 |
| **5.16** | Mijn organisatie heeft veel invloed op het beleid op het terrein van sport en recreatie in de regio. |  | 🞏 🞏 🞏 🞏 🞏 |  | 🞏 🞏 🞏 🞏 🞏 |
| **5.17** | Mijn organisatie heeft veel invloed op het veiligheidsbeleid in de regio. |  | 🞏 🞏 🞏 🞏 🞏 |  | 🞏 🞏 🞏 🞏 🞏 |
| **5.18** | Andere ontwikkeling die van invloed is op de bijdrage van mijn organisatie (indien aanwezig): |  |  |  | 🞏 🞏 🞏 🞏 🞏 |

Vermeld **in het algemeen** uw belangrijkste samenwerkingspartners als u kijkt naar het afgelopen decennium met een maximum van vijf **(kolom E)**. Geef vervolgens aan hoe intensief u met deze partner samenwerkt **(kolom F)** en hoe u groot de opbrengst van deze samenwerking is voor uw organisatie. **(kolom G)**

|  | **E**  Mijn belangrijkste samenwerkingspartners zijn: |  | zeer **F** geheel niet  intensief intensief  1 2 3 4 5 |  | hoge **G** lage  opbrengst opbrengst  1 2 3 4 5 |
| --- | --- | --- | --- | --- | --- |
| **6.1** |  |  | 🞏 🞏 🞏 🞏 🞏 |  | 🞏 🞏 🞏 🞏 🞏 |
| **6.2** |  |  | 🞏 🞏 🞏 🞏 🞏 |  | 🞏 🞏 🞏 🞏 🞏 |
| **6.3** |  |  | 🞏 🞏 🞏 🞏 🞏 |  | 🞏 🞏 🞏 🞏 🞏 |
| **6.4** |  |  | 🞏 🞏 🞏 🞏 🞏 |  | 🞏 🞏 🞏 🞏 🞏 |
| **6.5** |  |  | 🞏 🞏 🞏 🞏 🞏 |  | 🞏 🞏 🞏 🞏 🞏 |

**Bent u in het kader van schoolSlag gaan samenwerken met organisaties waarmee u voorheen geen samenwerkingscontacten onderhield?**

🞏 Nee

🞏 Ja, namelijk:

- …………………………………………………………………………………………………….…
- ……………………………………………………………………………………………………….
- ……………………………………………………………………………………………………….
- ……………………………………………………………………………………………………….
- ………………………………………………………………………………………………………

**Welke persoon (of personen) is (zijn) op dit moment de belangrijkste aanjager(s) van schoolSlag volgens u?**

- …………………………………………………………………………………………………….…
- ……………………………………………………………………………………………………….
- ……………………………………………………………………………………………………….
- ……………………………………………………………………………………………………….

**Welke persoon (of personen) mist u op dit moment als aanjager van schoolSlag?**

🞏 Geen

🞏 Ik mis:

- …………………………………………………………………………………………………….…
- ……………………………………………………………………………………………………….
- ……………………………………………………………………………………………………….
- ……………………………………………………………………………………………………….

In onderstaand overzicht treft u de huidige schoolSlag-samenwerkingspartners aan. Geef aan hoe intensief u zelf met deze partner samenwerkt in het kader van schoolslag **(kolom H)** en hoe hoog u opbrengst van deze specifieke samenwerking inschat voor uw eigen organisatie **(kolom I)**. De regel over uw eigen organisatie kunt u leeg laten.

**Nb.** Als u een of meerdere samenwerkingspartners in dit overzicht mist, kunt u deze zelf toevoegen.

|  | **Samenwerkingspartners in vraaggestuurd preventief jeugdbeleid :** |  | intensieve **H** geen directe  samenwerking samenwerking  1 2 3 4 5 |  | hoge **I** geen  opbrengst opbrengst  1 2 3 4 5 |
| --- | --- | --- | --- | --- | --- |
| **7.1** | Gemeente Maastricht |  | 🞏 🞏 🞏 🞏 🞏 |  | 🞏 🞏 🞏 🞏 🞏 |
| **7.2** | Overige Heuvelland gemeenten in gewest |  | 🞏 🞏 🞏 🞏 🞏 |  | 🞏 🞏 🞏 🞏 🞏 |
| **7.3** | Provincie Limburg |  | 🞏 🞏 🞏 🞏 🞏 |  | 🞏 🞏 🞏 🞏 🞏 |
| **7.4** | Landelijke overheid |  | 🞏 🞏 🞏 🞏 🞏 |  | 🞏 🞏 🞏 🞏 🞏 |
| **7.5** | Voorgezet Onderwijs |  | 🞏 🞏 🞏 🞏 🞏 |  | 🞏 🞏 🞏 🞏 🞏 |
| **7.6** | Basis Onderwijs / WSNS |  | 🞏 🞏 🞏 🞏 🞏 |  | 🞏 🞏 🞏 🞏 🞏 |
| **7.7** | Speciaal Voorgezet Onderwijs |  | 🞏 🞏 🞏 🞏 🞏 |  | 🞏 🞏 🞏 🞏 🞏 |
| **7.8** | Speciaal Basis Onderwijs |  | 🞏 🞏 🞏 🞏 🞏 |  | 🞏 🞏 🞏 🞏 🞏 |
| **7.9** | Bureau Jeugdzorg |  | 🞏 🞏 🞏 🞏 🞏 |  | 🞏 🞏 🞏 🞏 🞏 |
| **7.10** | CAD Limburg – instituut voor verslavingszorg |  | 🞏 🞏 🞏 🞏 🞏 |  | 🞏 🞏 🞏 🞏 🞏 |
| **7.11** | GGD Zuidelijk Zuid-Limburg |  | 🞏 🞏 🞏 🞏 🞏 |  | 🞏 🞏 🞏 🞏 🞏 |
| **7.12** | RIAGG Maastricht |  | 🞏 🞏 🞏 🞏 🞏 |  | 🞏 🞏 🞏 🞏 🞏 |
| **7.13** | Stichting Trajekt |  | 🞏 🞏 🞏 🞏 🞏 |  | 🞏 🞏 🞏 🞏 🞏 |
| **7.14** | Bureau Halt Maastricht Mergelland |  | 🞏 🞏 🞏 🞏 🞏 |  | 🞏 🞏 🞏 🞏 🞏 |
| **7.15** | Huis voor de Sport Limburg |  | 🞏 🞏 🞏 🞏 🞏 |  | 🞏 🞏 🞏 🞏 🞏 |
| **7.16** | Politie Limburg Zuid |  | 🞏 🞏 🞏 🞏 🞏 |  | 🞏 🞏 🞏 🞏 🞏 |
| **7.17** | Nederlands Instituut voor Gezondheidsbevordering en Preventie (NIGZ) |  | 🞏 🞏 🞏 🞏 🞏 |  | 🞏 🞏 🞏 🞏 🞏 |
| **7.18** |  |  | 🞏 🞏 🞏 🞏 🞏 |  | 🞏 🞏 🞏 🞏 🞏 |
| **7.19** |  |  | 🞏 🞏 🞏 🞏 🞏 |  | 🞏 🞏 🞏 🞏 🞏 |
| **7.20** |  |  | 🞏 🞏 🞏 🞏 🞏 |  | 🞏 🞏 🞏 🞏 🞏 |

Als u de huidige schoolSlag-samenwerking een rapportcijfer mag geven van 1 (zeer slecht) t/m 10 (uitmuntend), welk cijfer krijgt deze samenwerking van u?

. .

| Geef hier uw mening over de mate van belangrijkheid van de doelen van schoolSlag | | | | | | | | |
| --- | --- | --- | --- | --- | --- | --- | --- | --- |
|  |  | **Zeer**  **Belangrijk** | | | | **Geheel**  **onbelangrijk** | | |
| Hoe belangrijk vindt u het dat schoolSlag zich richt op: | | |  |  |  | |  |  |
| **8.1** | - het bevorderen van gezond gedrag in de leeftijdsgroep 0 –23; | | 🞏 | 🞏 | 🞏 | | 🞏 | 🞏 |
| **8.2** | - het terugdringen van probleemgedrag in de leeftijdsgroep 0-23; | | 🞏 | 🞏 | 🞏 | | 🞏 | 🞏 |
| **8.3** | - het functioneren als één loket voor het onderwijs; | | 🞏 | 🞏 | 🞏 | | 🞏 | 🞏 |
| **8.4** | - het vraaggestuurd werken ten behoeve van het onderwijs; | | 🞏 | 🞏 | 🞏 | | 🞏 | 🞏 |
| **8.5** | - het verhogen van de planmatigheid op het terrein van gezondheidsbevordering en preventie in, met en rondom het onderwijs; | | 🞏 | 🞏 | 🞏 | | 🞏 | 🞏 |
| **8.6** | - het realiseren van integrale samenwerking op het terrein van gezondheidsbevordering en preventie gericht op jeugd; | | 🞏 | 🞏 | 🞏 | | 🞏 | 🞏 |
| **8.7** | - het verhogen van de effectiviteit van gezondheidsbevordering en preventie; | | 🞏 | 🞏 | 🞏 | | 🞏 | 🞏 |
| **8.8** | - het ontdubbelen van gezondheidsbevorderings- en preventieaanbod; | | 🞏 | 🞏 | 🞏 | | 🞏 | 🞏 |
| **8.9** | - het bundelen van gezondheidsbevorderings- en preventie expertise. | | 🞏 | 🞏 | 🞏 | | 🞏 | 🞏 |
| **8.10** | Hoe belangrijk vindt u het dat *gezamenlijk* aan deze doelen gewerkt wordt? | | 🞏 | 🞏 | 🞏 | | 🞏 | 🞏 |
| **8.11** | Hoe belangrijk vindt u het dat deze aanpak een *blijvend* karakter krijgt? | | 🞏 | 🞏 | 🞏 | | 🞏 | 🞏 |
| **8.12** | Zijn er andere doelen waarop vraaggestuurd preventief jeugdbeleid zich zou moeten richten?  🞏 Nee  🞏 Ja, namelijk: | | | | | | | |

| Geef bij de volgende stellingen aan in hoeverre u het er mee eens bent. | | | | | | | | |
| --- | --- | --- | --- | --- | --- | --- | --- | --- |
|  | |  | geheel  oneens | | | geheel  eens | | |
| **9.1** | Ik vind het belangrijk dat mijn eigen organisatie meewerkt aan schoolSlag | | 🞏 | 🞏 | 🞏 | | 🞏 | 🞏 |
| **9.2** | Ik vind dat schoolSlag een belangrijke bijdrage levert aan de realisering van de doelstellingen van mijn eigen organisatie. | | 🞏 | 🞏 | 🞏 | | 🞏 | 🞏 |
| **9.3** | schoolSlag biedt nieuwe kansen voor mijn eigen organisatie. | | 🞏 | 🞏 | 🞏 | | 🞏 | 🞏 |
| **9.4** | De centrale doelen van mijn organisatie zijn volledig haalbaar zonder samenwerking met de schoolSlag-partners. | | 🞏 | 🞏 | 🞏 | | 🞏 | 🞏 |
| **9.5** | Op dit moment zijn voor mijn organisatie de voordelen van de schoolSlag-werkwijze groter dan de nadelen. | | 🞏 | 🞏 | 🞏 | | 🞏 | 🞏 |
| **9.6** | Op dit moment bestaan er knelpunten die eerst opgelost moeten worden voordat schoolSlag in mijn organisatie een succes kan zijn. | | 🞏 | 🞏 | 🞏 | | 🞏 | 🞏 |

Geef van de volgende stellingen aan in hoeverre u het er mee eens bent.

|  | | | geheel  oneens | | |  | | geheel eens | | |
| --- | --- | --- | --- | --- | --- | --- | --- | --- | --- | --- |
| **Binnen de schoolSlag-samenwerking ervaar ik dat:** | | |  |  |  | | | | | |
| **10.1** | - er duidelijk respect is voor ieders identiteit; | | 🞏 | 🞏 | 🞏 | | 🞏 | | 🞏 | |
| **10.2** | - er overeenstemming is over ieders rol (taak); | | 🞏 | 🞏 | 🞏 | | 🞏 | | 🞏 | |
| **10.3** | - er overeenstemming is over de afbakening van ieders werkterrein; | | 🞏 | 🞏 | 🞏 | | 🞏 | | 🞏 | |
| **10.4** | - er een duidelijke regie aanwezig is; | | 🞏 | 🞏 | 🞏 | | 🞏 | | 🞏 | |
| **10.5** | - de regie gevoerd wordt door de juiste partij. | | 🞏 | 🞏 | 🞏 | | 🞏 | | 🞏 | |
| **Binnen de schoolSlag-samenwerking ervaar ik dat er overeenstemming is over:** | | | **oneens eens** | | | | | | | |
| **11.1** | - het belang dat gehecht wordt aan preventie op het terrein van gezondheid, welzijn en veiligheid als onderdeel van integrale leerlingenzorg; | | 🞏 | 🞏 | 🞏 | | 🞏 | | | 🞏 |
| **11.2** | - het belang dat gehecht wordt een integrale ondersteuning (extern) van het onderwijs op het terrein van gezondheid / welzijn / veiligheid; | | 🞏 | 🞏 | 🞏 | | 🞏 | | | 🞏 |
| **11.3** | - het belang van regionale samenwerking ten behoeve van gezondheidsbevordering en preventie; | | 🞏 | 🞏 | 🞏 | | 🞏 | | | 🞏 |
| **11.4** | - de werkwijze vraaggestuurde preventie jeugd; | | 🞏 | 🞏 | 🞏 | | 🞏 | | | 🞏 |
| **11.5** | - het belang van het gezamenlijk (onderwijs – instellingen –lokale overheid) ontwikkelen en realiseren van vraaggestuurde preventie. | | 🞏 | 🞏 | 🞏 | | 🞏 | | | 🞏 |
| **Als ik kijk naar de samenstelling van de schoolSlag-partners vind ik dat:** | | **oneens eens** | | | | | | | | |
| **12.1** | - er te veel organisaties in schoolSlag samenwerken; | | 🞏 | 🞏 | 🞏 | | 🞏 | | | 🞏 |
| **12.2** | - er één of meer potentiële samenwerkingspartners worden gemist; | | 🞏 | 🞏 | 🞏 | | 🞏 | | | 🞏 |
| **12.3** | - de verschillen tussen de schoolSlag-partners effectief samenwerken belemmert; | | 🞏 | 🞏 | 🞏 | | 🞏 | | | 🞏 |
| **12.4** | - de expertise van de schoolSlag-partners voldoende divers is om van gecoördineerd, vraaggestuurd schoolgezondheidsbeleid een succes te maken; | | 🞏 | 🞏 | 🞏 | | 🞏 | | | 🞏 |
| **12.5** | - het ondersteuningsaanbod van de schoolSlag-partners voldoende divers is om scholen integraal te kunnen ondersteunen op het terrein van gezondheidsbevordering en preventie. | | 🞏 | 🞏 | 🞏 | | 🞏 | | | 🞏 |

| Geef van de volgende stellingen aan in hoeverre u het er mee eens bent.. Als u over een item geen mening heeft omdat dit bijvoorbeeld onbekend is, vult u niets in. | | | | | | |
| --- | --- | --- | --- | --- | --- | --- |
|  |  | geheel oneens |  |  | geheel  eens | |
| **13.1** | De schoolSlag-samenwerking vindt plaats in een sfeer van concurrentie. | 🞏 | 🞏 | 🞏 | 🞏 | 🞏 |
| **13.2** | De schoolSlag-partners zijn competent voor hun taken. | 🞏 | 🞏 | 🞏 | 🞏 | 🞏 |
| **13.3** | De schoolSlag-parnters zijn volledig te vertrouwen. | 🞏 | 🞏 | 🞏 | 🞏 | 🞏 |
| **13.4** | Onderlinge afspraken in het kader van schoolSlag worden altijd stipt nagekomen. | 🞏 | 🞏 | 🞏 | 🞏 | 🞏 |
| **13.5** | De ontwikkeling en realisatie van schoolSlag is een taak van mijn eigen organisatie. | 🞏 | 🞏 | 🞏 | 🞏 | 🞏 |
| **13.6** | Mijn collega’s zijn positief over de ontwikkeling van schoolSlag. | 🞏 | 🞏 | 🞏 | 🞏 | 🞏 |
| **13.7** | Mijn collega’s zien schoolSlag als een werkwijze van de eigen organisatie. | 🞏 | 🞏 | 🞏 | 🞏 | 🞏 |
| **13.8** | SchoolSlag is mede iets van mij. | 🞏 | 🞏 | 🞏 | 🞏 | 🞏 |
| **13.9** | Als schoolSlag aanpassingen vraagt van de interne organisatie van mijn organisatie dan sta ik daar open voor. | 🞏 | 🞏 | 🞏 | 🞏 | 🞏 |
| **13.10** | Ik heb zelf invloed op de aanpassingen in mijn eigen organisatie ten behoeve van schoolSlag, indien nodig. | 🞏 | 🞏 | 🞏 | 🞏 | 🞏 |
| **13.11** | Er zijn nu al zaken die ik in mijn eigen organisatie wil veranderen ten behoeve van schoolSlag. | 🞏 | 🞏 | 🞏 | 🞏 | 🞏 |
| **13.12** | Voor zover ik nu kan overzien vraagt schoolSlag geen enkele aanpassing van mijn organisatie. | 🞏 | 🞏 | 🞏 | 🞏 | 🞏 |

In dit blok stellen we vragen over de door uw organisatie ondernomen acties ten behoeve van de ontwikkeling en realisatie van schoolSlag.

| Geef daarom van de volgende stellingen aan of u het ermee eens bent of niet. | | | | | | |
| --- | --- | --- | --- | --- | --- | --- |
|  |  | **geheel**  **oneens** | |  | **geheel**  **eens** | |
| **14.1** | Ten behoeve van schoolSlag is in mijn organisatie de interne overleg/communicatie structuur aangepast. | 🞏 | 🞏 | 🞏 | 🞏 | 🞏 |
| **14.2** | T.b.v. schoolSlag heeft mijn organisatie productieafspraken aangepast in overleg met de opdrachtgever / financier. | 🞏 | 🞏 | 🞏 | 🞏 | 🞏 |
| **14.3** | Materialen/projecten van mijn organisatie t.b.v. gezondheidsbevordering en preventie zijn aangepast onder invloed van de schoolSlag-werkwijze. | 🞏 | 🞏 | 🞏 | 🞏 | 🞏 |
| **14.4** | Ten behoeve van schoolSlag is de verdeling van werkzaamheden in mijn organisatie aangepast. | 🞏 | 🞏 | 🞏 | 🞏 | 🞏 |
| **14.5** | Mijn organisatie heeft andere aanpassingen gemaakt ten behoeve schoolSlag, dan hiervoor genoemd, namelijk:   - ……………………………………………………………………………… - ……………………………………………………………………………… - ……………………………………………………………………………… |  |  |  |  |  |
| **14.6** | Mijn organisatie investeert in schoolSlag via de inzet van middelen. | 🞏 | 🞏 | 🞏 | 🞏 | 🞏 |
| **14.7** | Mijn organisatie investeert in schoolSlag via de inzet van menskracht. | 🞏 | 🞏 | 🞏 | 🞏 | 🞏 |
| **14.8** | De (beleids)doelen van mijn organisatie zijn aangepast onder invloed van schoolSlag. | 🞏 | 🞏 | 🞏 | 🞏 | 🞏 |
| **14.9** | Samenwerkingsafspraken in het kader van schoolSlag op hoger managementniveau (bestuur/directie) zijn vastgelegd. | 🞏 | 🞏 | 🞏 | 🞏 | 🞏 |
| **14.10** | Ten behoeve van schoolSlag door mijn organisatie één of meerdere nieuwe preventiemogelijkheden ontwikkeld,  🞏 Nee  🞏 Ja, namelijk:   - ……………………………………………………………………………… - ……………………………………………………………………………… - …………………………………………………………………………………… | |  |  |  |  |

Onderstaande stellingen gaan over de wijze waarop schoolSlag in de regio gestalte krijgt. Als u geen antwoord weet omdat iets u onbekend is, vult u niets in.

|  | |  | **geheel**  **oneens** | | **geheel**  **eens** | | | |
| --- | --- | --- | --- | --- | --- | --- | --- | --- |
| **15.1** | De ontwikkeling van schoolSlag wordt goed doordacht. | | 🞏 | 🞏 | | 🞏 | 🞏 | 🞏 |
| **15.2** | De ontwikkeling van schoolSlag gebeurt planmatig. | | 🞏 | 🞏 | | 🞏 | 🞏 | 🞏 |
| **15.3** | schoolSlag voorziet in een vraag vanuit het onderwijsveld. | | 🞏 | 🞏 | | 🞏 | 🞏 | 🞏 |
| **15.4** | Keuzes in het ontwikkelproces van schoolSlag worden voldoende besproken in relevante overleggen. | | 🞏 | 🞏 | | 🞏 | 🞏 | 🞏 |
| **15.5** | De schoolSlag-ontwikkeling wordt vooral gebaseerd op wetenschappelijke kennis. | | 🞏 | 🞏 | | 🞏 | 🞏 | 🞏 |
| **15.6** | De schoolSlag-ontwikkeling wordt vooral gebaseerd op praktijkkennis. | | 🞏 | 🞏 | | 🞏 | 🞏 | 🞏 |
| **15.7** | Medewerkers hebben meer invloed op de ontwikkeling van schoolSlag dan hun managers. | | 🞏 | 🞏 | | 🞏 | 🞏 | 🞏 |
| **15.8** | Op basis van inhoudsdeskundigheid wordt de schoolSlag werkwijze gericht aan de man gebracht. | | 🞏 | 🞏 | | 🞏 | 🞏 | 🞏 |
| **15.9** | Lokaal beleid is het meest bepalend voor het slagen van de schoolSlag werkwijze. | | 🞏 | 🞏 | | 🞏 | 🞏 | 🞏 |
| **15.10** | De schoolSlag-werkwijze wordt samen met het onderwijs ontwikkeld. | | 🞏 | 🞏 | | 🞏 | 🞏 | 🞏 |
| **15.11** | Partners ontwikkelen zelf eigen werkvormen voor het toepassen van de schoolSlag-werkwijze. | | 🞏 | 🞏 | | 🞏 | 🞏 | 🞏 |
| **15.12** | De coördinator is overtuigend als het gaat om de noodzaak om gezamenlijk te investeren in gezondheidsbevordering en preventie. | | 🞏 | 🞏 | | 🞏 | 🞏 | 🞏 |
| **15.13** | Het coördinator behandelt alle schoolSlag-partners met vertrouwen en respect. | | 🞏 | 🞏 | | 🞏 | 🞏 | 🞏 |
| **15.14** | De manier waarop ik geïnformeerd wordt over schoolSlag vind ik helder en voldoende. | | 🞏 | 🞏 | | 🞏 | 🞏 | 🞏 |
| **15.15** | Het coördinator maakt het mogelijk dat ik zelf de ontwikkeling van schoolSlag kan beïnvloeden. | | 🞏 | 🞏 | | 🞏 | 🞏 | 🞏 |
| **15.16** | Mijn bijdrage via persoonlijk contact en/of tijdens gezamenlijke discussies heeft de schoolSlag-samenwerking beïnvloed. | | 🞏 | 🞏 | | 🞏 | 🞏 | 🞏 |
| **15.17** | De coördinator zorgt er voor dat mijn persoonlijk netwerk onderhouden en waar nodig verbreed wordt t.b.v. schoolSlag. | | 🞏 | 🞏 | | 🞏 | 🞏 | 🞏 |
| **15.18** | De coördinator onderhoudt een relevant persoonlijk netwerk voor schoolSlag | | 🞏 | 🞏 | | 🞏 | 🞏 | 🞏 |

Wat is er in het kader van schoolSlag tot op heden in uw ogen bereikt? Ook hier geldt, is een item u volstrekt onbekend, vul dan niets in.

|  |  | **geheel oneens** |  |  | **geheel**  **eens** | |
| --- | --- | --- | --- | --- | --- | --- |
| **16.1** | schoolSlag heeft een duidelijk herkenbare plaats verworven in het preventieveld. | 🞏 | 🞏 | 🞏 | 🞏 | 🞏 |
| **16.2** | schoolSlag heeft een duidelijk herkenbare plaats verworven in het onderwijs. | 🞏 | 🞏 | 🞏 | 🞏 | 🞏 |
| **16.3** | schoolSlag heeft een duidelijk herkenbare plaats verworven in lokaal / regionaal beleid. | 🞏 | 🞏 | 🞏 | 🞏 | 🞏 |
| **16.4** | schoolSlag heeft door activiteiten in, met en rondom het onderwijs inhoud gegeven aan het bevorderen van ‘gezond leven’ in onze regio. | 🞏 | 🞏 | 🞏 | 🞏 | 🞏 |
| **16.5** | schoolSlag draagt merkbaar bij aan een betere samenwerking op het terrein van gezondheidsbevordering en preventie. | 🞏 | 🞏 | 🞏 | 🞏 | 🞏 |
| **16.6** | Scholen gaan bewuster om met gezondheidsbevordering en preventie als integraal onderdeel van hun zorgbeleid. | 🞏 | 🞏 | 🞏 | 🞏 | 🞏 |
| **16.7** | Wat is/zijn volgens u de belangrijkste (nog niet genoemde) knelpunten op dit moment voor schoolSlag? | | | | | |

De volgende stellingen betreffen uw meningen over elementen van schoolSlag.

|  | |  | **geheel**  **oneens** | | | | **geheel eens** |
| --- | --- | --- | --- | --- | --- | --- | --- |
| **17.1** | Onderwijsprofessionals zijn goed in staat geheel zelf hun prioriteiten op het terrein van gezondheidsbevordering en preventie te formuleren. | | 🞏 | 🞏 | 🞏 | 🞏 | 🞏 |
| **17.2** | Schoolprofielen met objectieve cijfers over de gezondheidsstatus van de schoolpopulatie zijn belangrijk voor vraagverheldering. | | 🞏 | 🞏 | 🞏 | 🞏 | 🞏 |
| **17.3** | Ouderparticipatie is belangrijk bij het stellen van prioriteiten op het terrein van gezondheidsbevordering en preventie in het onderwijs. | | 🞏 | 🞏 | 🞏 | 🞏 | 🞏 |
| **17.4** | Aanbieders moeten hun eigen preventie-aanbod enkel via schoolSlag bij het onderwijs onder de aandacht brengen. | | 🞏 | 🞏 | 🞏 | 🞏 | 🞏 |
| **17.5** | De huidige opzet van schoolSlag is identiek aan de ideeën die er bestonden voorjaar 2002 (start schoolSlag-coördinator). | | 🞏 | 🞏 | 🞏 | 🞏 | 🞏 |
| **17.6** | De schoolSlag-samenwerking tussen ondersteuningsorganisaties en gemeenten is onderdeel van gemeentelijke jeugdbeleid. | | 🞏 | 🞏 | 🞏 | 🞏 | 🞏 |
| **17.7** | Het beschikbaar stellen van objectieve cijfers over gezondheid, welzijn en veiligheid per school is een lokale verantwoordelijkheid. | | 🞏 | 🞏 | 🞏 | 🞏 | 🞏 |

Om vergelijkingen met andere informatiebronnen mogelijk te maken, vragen wij u hier een aantal persoonlijke gegevens in te vullen.

A. Naam: ………………………………………….

B. Organisatie: …………………………………..

C. Wat is uw huidige functie?

In het onderwijs

- Leerkracht
- Manager in het onderwijs
- Zorgcoördinator
- Lid werkgroep gezonde/veilige school

**Ondersteunende instelling**

- Uitvoerend medewerker getraind in het toepassen van vraaggestuurd preventief jeugdbeleid
- Uitvoerend medewerker (o.a. welzijnswerker, GVO-functionaris, preventiewerker), zonder vraaggestuurd preventief jeugdbeleid in takenpakket
- Leidinggevende
- Directielid

**Overheid**

- Ambtenaar Onderwijs
- Ambtenaars Welzijn / Gezondheid
- Leidinggevende Onderwijs
- Leidinggevende Welzijn/Gezondheid
- Wethouder met in portefeuille:…………………………………..

E. Hoeveel jaar relevante werkervaring heeft u?

- Meer dan 20 jaar
- Tussen de 10-20 jaar
- Tussen de 5 en 10 jaar
- Tussen de 2 en 5 jaar
- Minder dan 2 jaar

| **Ruimte voor algemene opmerkingen, tips & adviezen:** |  |  |  |  |  |
| --- | --- | --- | --- | --- | --- |


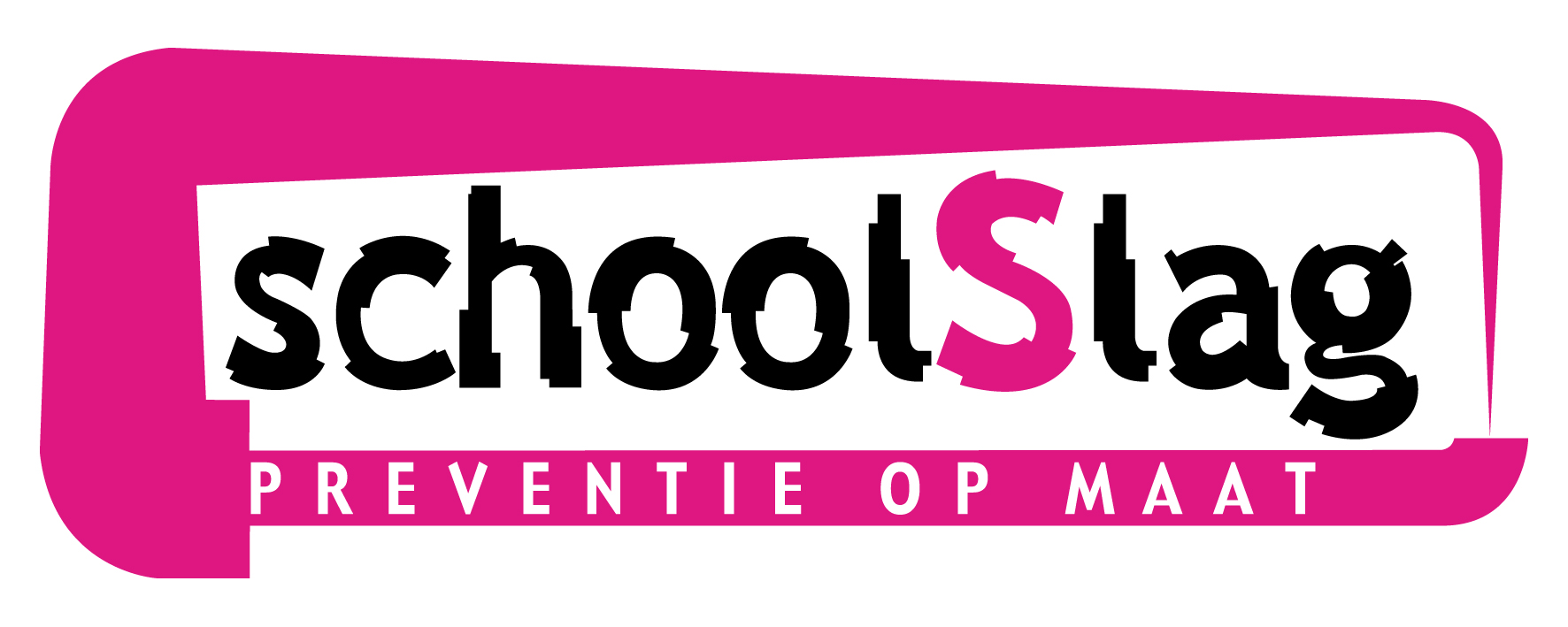


Hartelijk dank

voor het invullen!
